# Supplementary material for: Interferon restores replication fork stability and cell viability in BRCA-defective cells via ISG15
Source: Nat Commun. 2023 Oct 2;14:6140. doi: 10.1038/s41467-023-41801-w (PMC10545780; doi:10.1038/s41467-023-41801-w)
Supplement: Supplementary file 1 — Supplementary Information [file 41467_2023_41801_MOESM1_ESM.pdf]

Interferon restores replication fork stability and cell viability in BRCA-defective cells via ISG15

Ramona N. Moro, Uddipta Biswas, Suhas S. Kharat, Filip Duzanic, Prosun Das, Maria Stavrou, Maria C. Raso, Raimundo Freire, Arnab Ray Chaudhuri, Shyam K. Sharan, Lorenza Penengo

Supplementary Information

Supplementary Figures 1-6
Supplementary Methods including Supplementary Tables 1 and 2

Supplementary Figure 1.

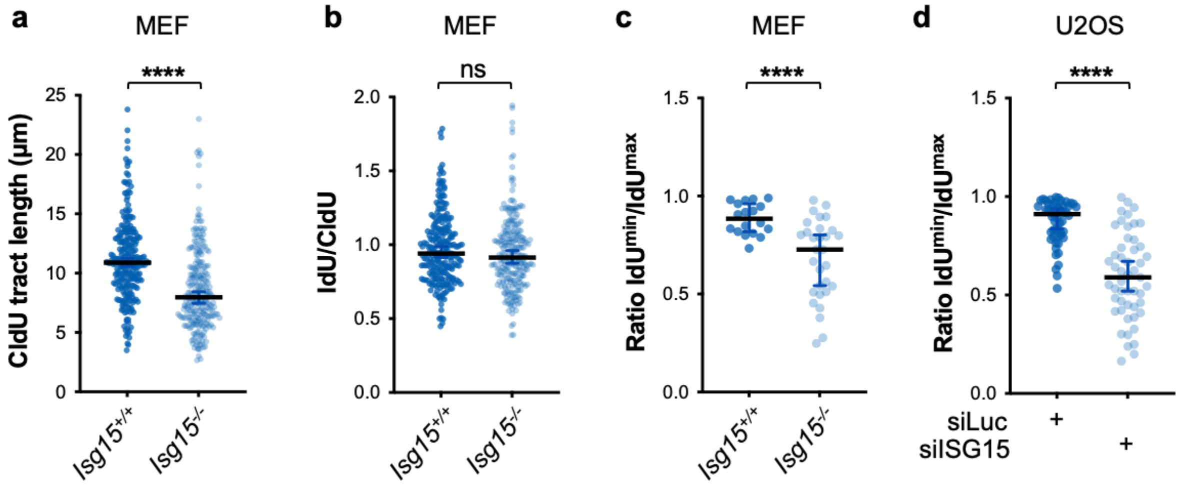

Supplementary Figure 1. Loss of ISG15 results in DNA replication fork slow down and sister fork asymmetry.

(**a, b**) Size distribution of CldU tract length measurements and IdU/CldU ratios in *Isg15*$^{+/+}$ and *Isg15*$^{-/-}$ MEFs from three independent experiments (*Isg15*$^{+/+}$ n = 240 and *Isg15*$^{-/-}$ n = 243). corresponding to Fig. **1c**. (**c, d**) Sister fork symmetry analysis in parental (*Isg15*$^{+/+}$), *Isg15*$^{-/-}$ MEFs (n = 3), and siLuc or siISG15 treated U2OS cells from three independent experiments (*Isg15*$^{+/+}$ n = 18 and *Isg15*$^{-/-}$ n = 30; siLuc n = 53 and siISG15 n = 51), displayed as a ratio of the shorter IdU tract to the longer IdU tract of the same sister fork. **a-d** Median value with 95% CI is shown. Two-tailed Mann-Whitney test was performed; ****, P<0.0001. Source data are provided as a Source Data file.

Supplementary Figure 2.

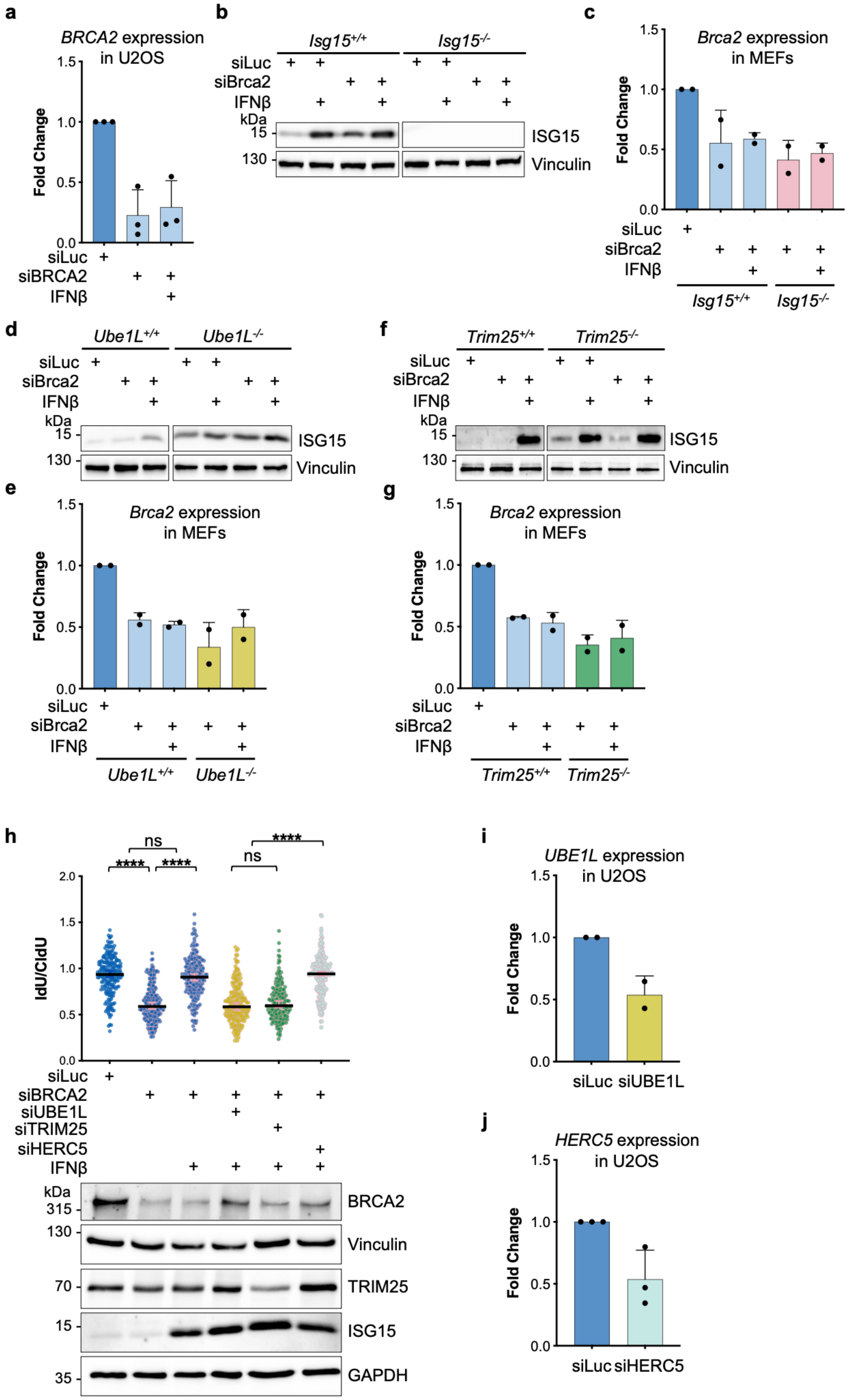

Supplementary Figure 2. IFNβ and ISG15 restore fork protection in BRCA-deficient cells via ISG15 conjugation.

(**a**) *BRCA2* mRNA expression in siBRCA2 U2OS cells treated with ± IFNβ (30 U/mL, 2 h) and chased for 46 h (n = 3), measured by qPCR and corresponding to Fig. **2b, c**. (**b**) Immunoblot showing levels of ISG15 expression in parental (*Isg15*$^{+/+}$) and *Isg15*$^{-/-}$ MEF cells with IFNβ treatment corresponding to Fig. **3b**. Vinculin, loading control. (**c**) *Brca2* mRNA expression in parental (*Isg15*$^{+/+}$) and *Isg15*$^{-/-}$ MEF cells measured by qPCR (n = 2) corresponding to Fig. **3b**.
(**d**) Immunoblot showing levels of ISG15 expression in parental (*Ube1L*$^{+/+}$) and *Ube1L*$^{-/-}$ MEF cells with IFNβ treatment corresponding to Fig. **3f**. Vinculin, loading control. (**e**) *Brca2* mRNA expression in parental (*Ube1L*$^{+/+}$) and *Ube1L*$^{-/-}$ MEF cells measured by qPCR (n = 2) corresponding to Fig. **3f**.
(**f**) Immunoblot showing levels of ISG15 expression in parental (*Trim25*$^{+/+}$) and *Trim25*$^{-/-}$ MEF cells with IFNβ treatment corresponding to Fig. **3g**. Vinculin, loading control. (**g**) *Brca2* mRNA expression in parental (*Trim25*$^{+/+}$) and *Trim25*$^{-/-}$ MEF cells measured by qPCR (n = 2) corresponding to Fig. **3g**.
(**h**) IdU/CldU ratio analysis from two independent experiments for the indicated conditions (siLuc n = 209, siBRCA2 n = 203, siBRCA2 +IFNβ n = 205, siUBE1L + siBRCA2 +IFNβ n = 206, siTRIM25 + siBRCA2 +IFNβ n = 209 and siHERC5 + siBRCA2 +IFNβ n = 208) along with BRCA2, TRIM25 and ISG15 protein expression. Median value with 95% CI is shown. Two-tailed Kruskal-Wallis test was performed; ****, P<0.0001. (**I, j**) *UBE1L* (n = 2) and *HERC5* (n = 3) expression in U2OS cells upon indicated siRNA treatment measured by qPCR and corresponding to Supplementary Fig. **1h**. **a,c,e,g,i** Data are represented as mean + SD. Source data are provided as a Source Data file.

Supplementary Figure 3.

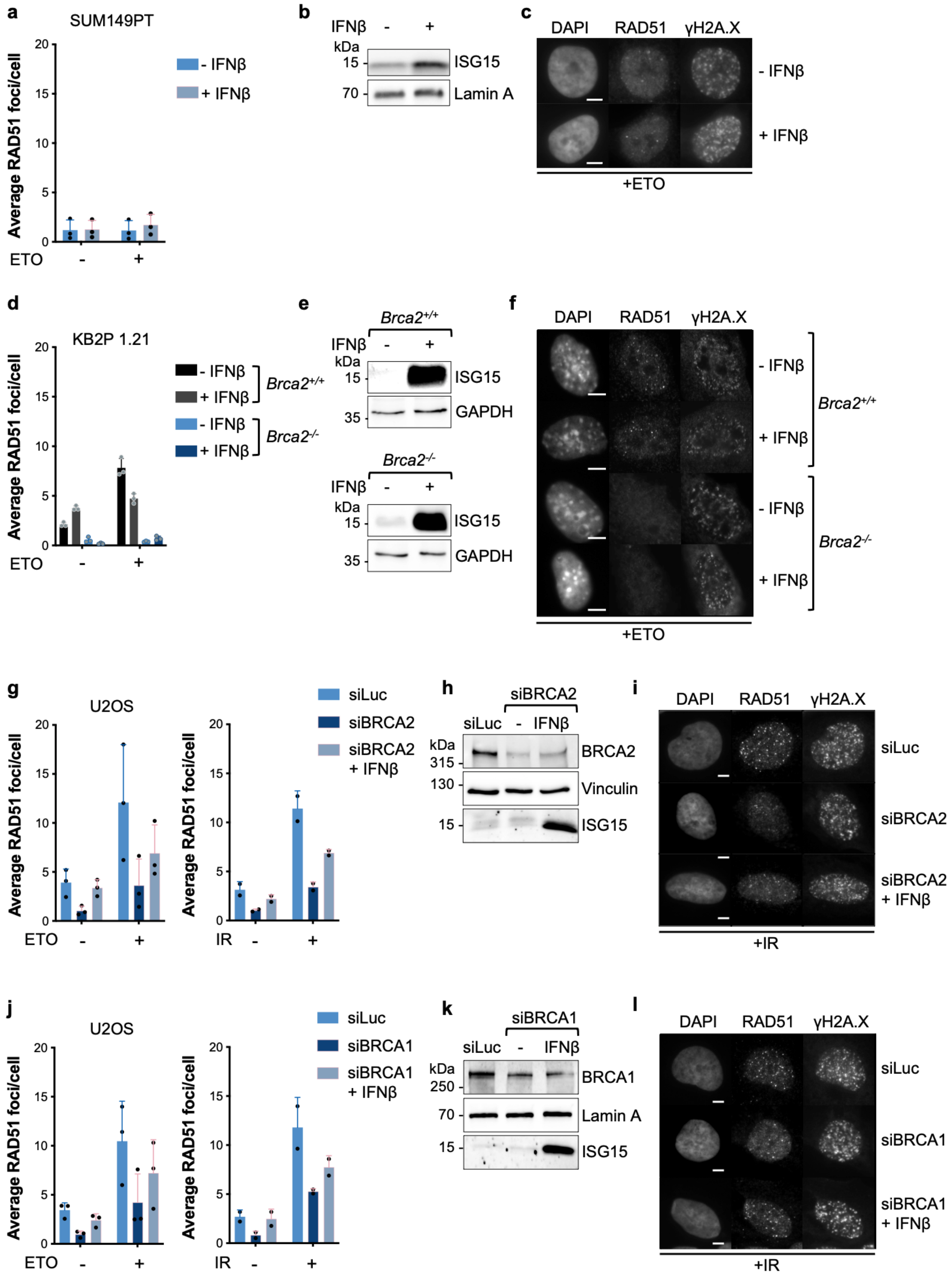

Supplementary Figure 3. IFNβ and ISG15 do not restore homologous recombination in BRCA-deficient cells.

(**a**) Average number of RAD51 foci per cell in SUM149PT cells upon ± IFNβ (30 U/mL, 2 h; 46 h chase). Cells were optionally treated with 5 μM etoposide (ETO) for 1 h and subsequently stained with antibodies against RAD51 and γH2AX. Data are represented as mean + SD (n = 3). (**b, c**) ISG15 expression and representative images of RAD51 foci formation in SUM149PT cells as in **a**. Lamin A, loading control. Scale bars, 5 μm. (**d**) Average number of RAD51 foci in KB2P cells (*Brca2*$^{+/+}$ and *Brca2*$^{-/-}$) treated with ± IFNβ (30 U/mL, 2h) and chased for 46h. Cells were treated with 5 μM of etoposide for 1 h and subsequently stained with antibodies against RAD51 and γH2AX. Data are represented as mean + SD (n = 3). (**e, f**) ISG15 expression and representative images of RAD51 foci in KB2P cells as in **d**. GAPDH, loading control. Scale bars, 5 μm. (**g**) Average number of RAD51 foci per cell in untreated, ETO-treated (n = 3) or irradiated (n = 2; 4 Gray) siBRCA2 or siLuc U2OS cells treated with ± IFNβ (30 U/mL, 2 h; 46 h chase). One hour after irradiation cells were fixed and subsequently stained with antibodies against RAD51 and γH2AX. Data are represented as mean + SD. (**h, i**) BRCA2 and ISG15 expression and representative images of RAD51 foci in U2OS cells as in **g**. Vinculin, loading control. Scale bars, 5 μm. (**j**) Average number of RAD51 foci per cell in untreated, ETO-treated (n = 3) or irradiated (n = 2; 4 Gray) siBRCA1 or siLuc U2OS cells treated with ± IFNβ (30 U/mL, 2 h; 46 h chase). One hour after irradiation cells were fixed and subsequently stained with antibodies against RAD51 and γH2AX. Data are represented as mean + SD. (**k, l**) BRCA1 and ISG15 expression and representative images of RAD51 foci in U2OS cells as in **j**. Lamin A, loading control. Scale bars, 5 μm. Source data are provided as a Source Data file.

Supplementary Figure 4.

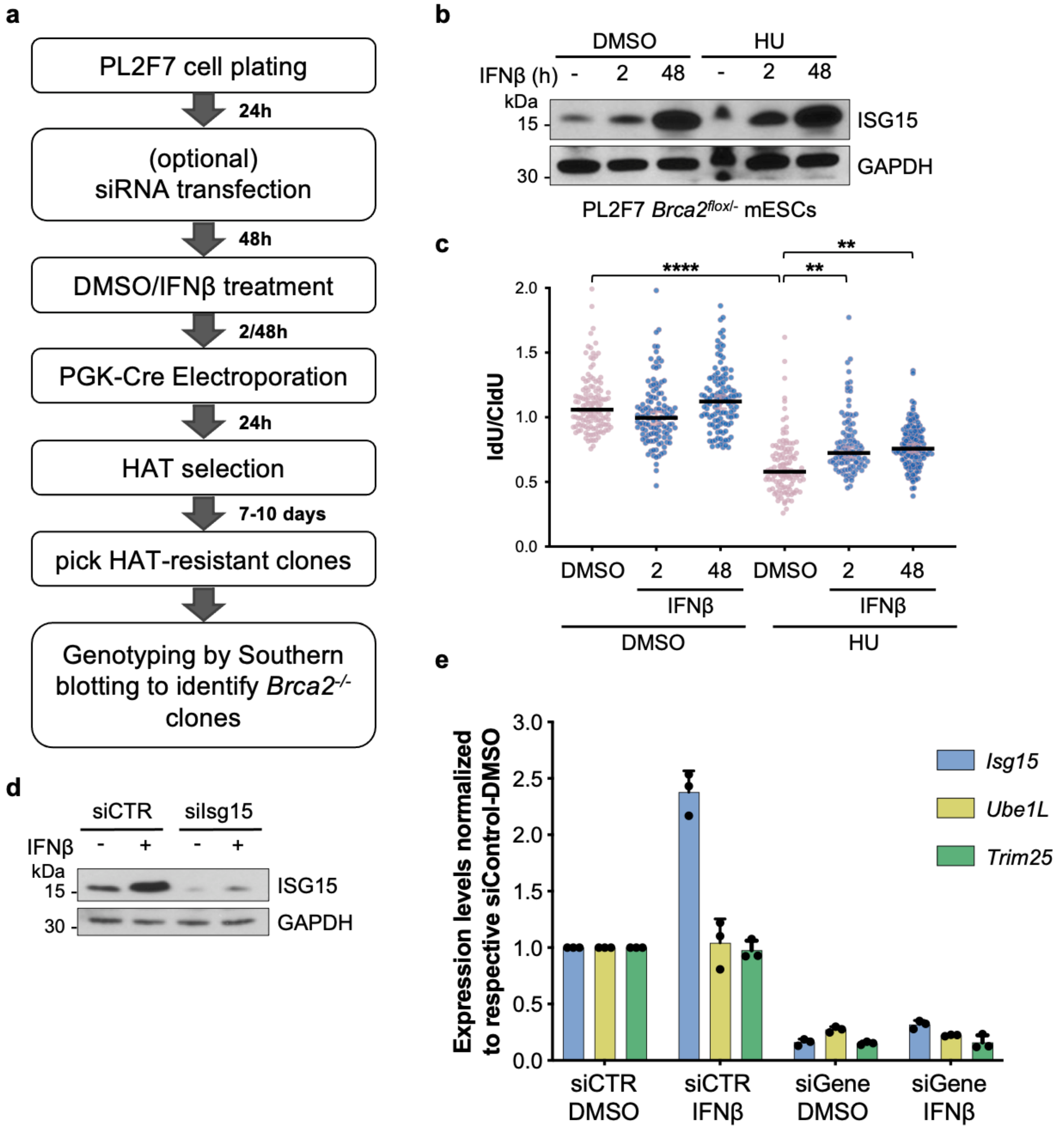

Supplementary Figure 4. Upregulation of IFNβ/ISG15 system restores viability in BRCA2-deficient mESCs.

(**a**) Schematic representation of the experimental workflow to test the effect of the IFN system on the viability of *Brca2*$^{-/-}$ mESCs. (**b**) Immunoblot showing the levels of ISG15 upon induction with ± IFNβ (30 U/mL, 2 h) pre-treatment and depletion by siRNA. GAPDH, loading control. (**c**) IdU/CldU ratio analysis in the hypomorphic PL2F7-*Brca2*$^{-/-}$;BRCA2 (R2336H) cells treated with ± IFNβ (30 U/mL, 2 h; 46 h chase) (DMSO n = 126, 2h IFNβ n = 121, 48h IFNβ n = 120, DMSO +HU n = 111, 2h IFNβ +HU n = 109 and 48h IFNβ +HU n = 137). Median value with 95% CI is shown. Two-tailed Kruskal-Wallis test was performed; **, P = 0.0011, ****, P<0.0001. (**d**) Immunoblot showing ISG15 protein levels upon IFNβ (30 U/mL, 2h) pre-treatment along with either DMSO or with hydroxyurea (HU, 4 mM) for 3 h. (**e**) Expression levels of *Isg15*, *Ube1L* and *Trim25* in mESC with ± IFNβ (30 U/mL, 2 h) pre-treatment and depletion by indicated siRNA. Data are represented as mean + SD. Source data are provided as a Source Data file.

Supplementary Figure 5.

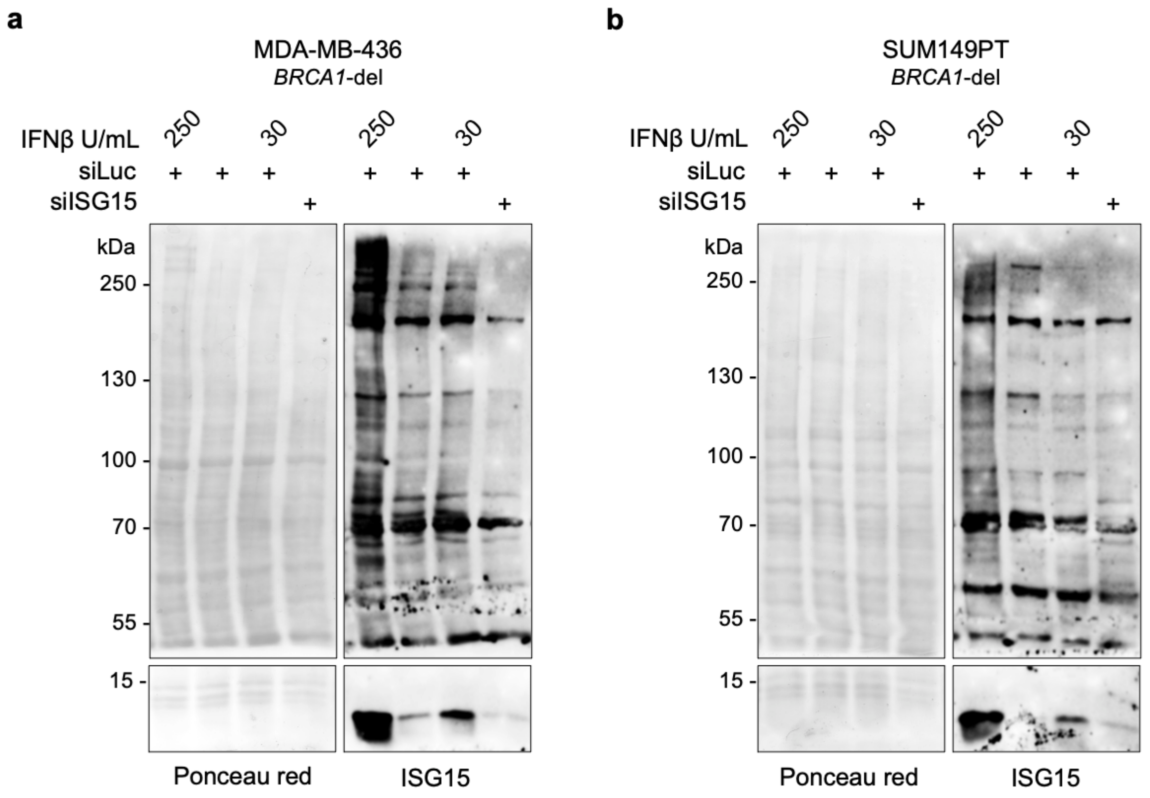

Supplementary Figure 5. Treatment with low dose of IFNβ does not induce detectable formation of ISG15 conjugates.

(**a, b**) Immunoblot showing the expression of ISG15 and the formation of ISGylated conjugates in siLuc or siISG15 MDA-MB-436 and SUM149PT cells, treated with high (250 U/mL) or low (30 U/mL) doses of IFNβ (2 h and 46 h chase). Ponceau red shows equal loading. Source data are provided as a Source Data file.

Supplementary Figure 6.

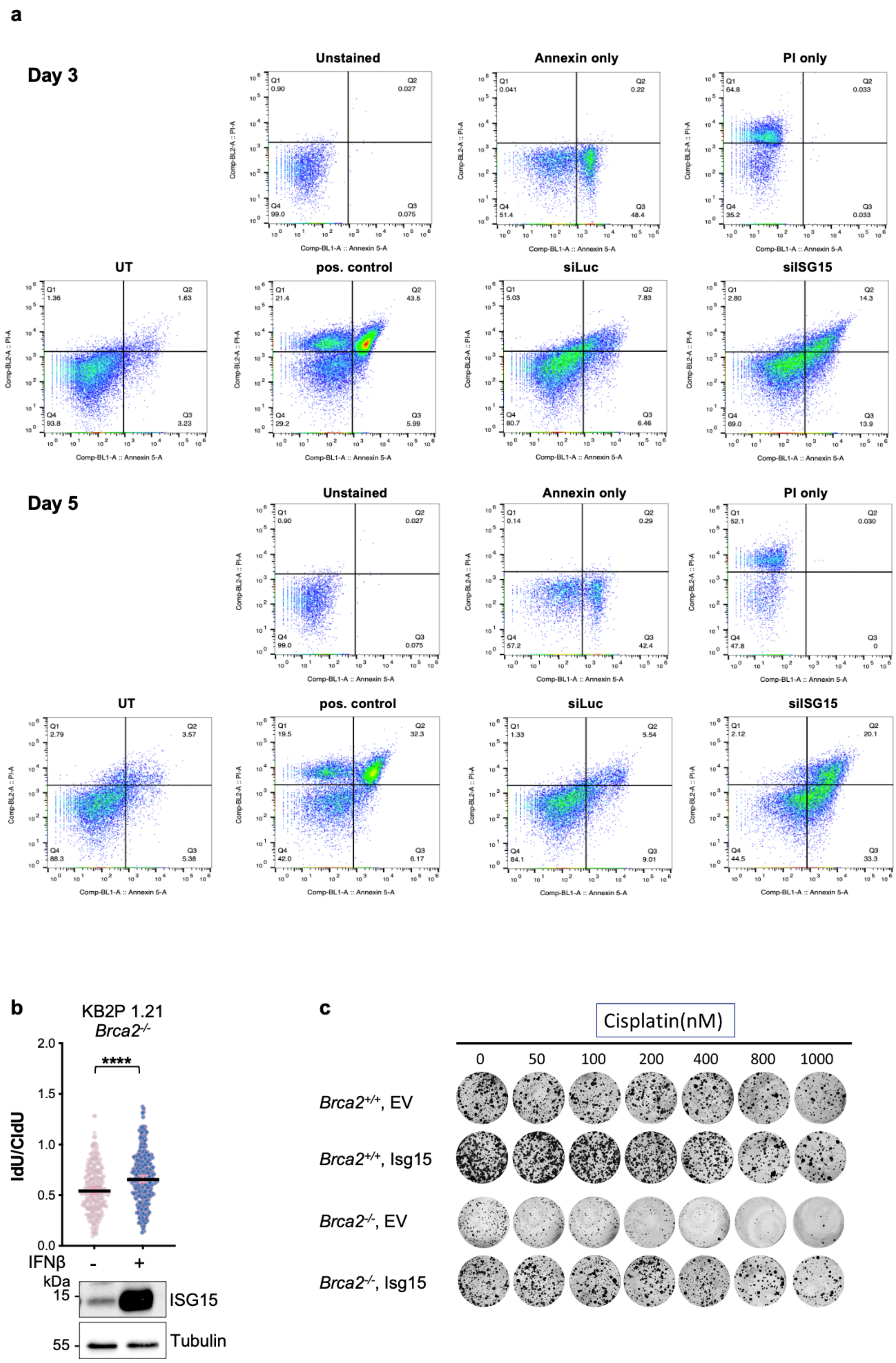

Supplementary Figure 6. ISG15 loss induces cell death in MDA-MB-436 cells, and its upregulation promotes fork protection and reduces drug sensitivity in KB2P cells.

(**a**) FACS analysis at day 3 and 5 upon siISG15 or siLuc transfected cells. Cell death was measured by using propidium iodide (PI) and Annexin V, to assess necrotic and apoptotic cells, respectively. As positive control, we treated cells at 55°C for 20 min and then mixed with untreated cells. We observed that at day 5 after ISG15 depletion the majority of cells are apoptotic, while in siLuc-transfected cells up to 84% of cells are viable. (**b**) IdU/CldU ratio analysis in *Brca2*$^{-/-}$ *p53*$^{-/-}$ KB2P cells treated with ± IFNβ (30 U/mL, 2 h; 46 h chase) from three independent experiments (-IFNβ n = 256 and +IFNβ n = 435) along with ISG15 protein expression. Horizontal lines represent the median value. Statistical analysis according to Mann-Whitney test was performed; ns, non-significant; ****, P<0.0001. (**c**) Representative colony images of the clonogenic assay as in **6e** upon increasing doses of Cisplatin. Source data are provided as a Source Data file.

Supplementary Methods

Supplementary Table 1. List of antibodies

Antibody	Source	Cat	RRID	Dilution
BRCA1	Santa Cruz Biotechnology	sc-6954	AB_626761	1:50
BRCA2	Millipore	OP95-100ug	AB_213443	1:500
FLAG	Sigma	F7425	AB_439687	1:1000
GAPDH	Millipore	MAB374	AB_2107445	1:50'000
ISG15	This paper	N/A	N/A	1:1000
ISG15	K.P. Knobeloch Institute of Neuropathology, Freiburg, Germany	N/A	N/A	1:1000
ISG15	Santa Cruz Biotechnology	sc-166755	AB_2126308	1:1000
Lamin A	Sigma-Aldrich	L1293	AB_532254	1:1000
MYC	Santa Cruz Biotechnology	sc-40	AB_627268	1:1000
PCNA	Santa Cruz Biotechnology	sc-56	AB_628110	1:1000
TOP1	Novus Biologicals	NBP1-90365	AB_11023377	1:2000
TRIM25	This paper	N/A	N/A	1:1000
TRIM25	Abcam	ab167154	AB_2721902	1:2000
Tubulin	Sigma-Aldrich	T5168	AB_477579	1:8000
Vinculin	Thermo Fisher Scientific	700062	AB_2532280	1:1000

Supplementary Table 2. qPCR primers (LightCycler® SYBR Green system):

Human *UBE1L*

forward: 5’- TGATGTTTGAGAAGGATGATG -3’

reverse: 5'- CCGGTGGAATCCCGTAGTT -3'

Human *GAPDH*

forward: 5’- ACAACTTTGGTATCGTGGAAG -3’

reverse: 5'- GCCATCACGCCACAGTTTC -3'

Mouse *Isg15*

forward: 5’-GTGGTACAGAACTGCAGCGA-3’

reverse: 5'-TCAGCCAGAACTGGTCTTCG-3'

Mouse *Ube1L*

forward: 5’- GGAGTTAGGGCGAATGGAGG -3’

reverse: 5'-GGAGTTAGGGCGAATGGAGG -3'

Mouse *Trim25*

forward: 5’- ATGGCTCAGGTAACAAGGGAG -3’

reverse: 5'- GGGAGCAACAGGGGTTTTCTT -3'

Mouse *Brca2*

forward:5’- AGCCCAGCTTGAAGCAAGT -3’

reverse:5'- GGATCATTCGGTAAACAGCG -3'

Mouse *Actin*

forward: 5’- CTGTCCCTGTATGCCTCTG -3’

reverse: 5'- ATGTCACGCACGATTTCC -3'

TaqMan® Gene expression assays:

Human *HERC5*: Hs00180943_m1

Human *BRCA2*: Hs00609073_m1

Human *Actin*: Hs01060665_g1

Mouse *Brca2*: Mm00464783_m1

Mouse *Hprt*: Mm03024075_m1
