# Supplementary material for: Interferon restores replication fork stability and cell viability in BRCA-defective cells via ISG15
Source: Nat Commun. 2023 Oct 2;14:6140. doi: 10.1038/s41467-023-41801-w (PMC10545780; doi:10.1038/s41467-023-41801-w)
Supplement: Supplementary file 3 — Description of Additional Supplementary Files [file 41467_2023_41801_MOESM3_ESM.pdf]

Description of Additional Supplementary Files

File Name: Supplementary Data 1
Description: this file contains the results of the mass spectrometry analysis on the chromatin composition of SUM149PT cells EV and ISG15 upon optional treatment with HU. The different sheets include all the hits identified, the significant hits, those enriched in EV or ISG15 upon HU treatment, and a comparison of factors obtained in untreated versus HU treated cells upon ISG15 induction.
