# Supplementary material for: Interferon restores replication fork stability and cell viability in BRCA-defective cells via ISG15
Source: Nat Commun. 2023 Oct 2;14:6140. doi: 10.1038/s41467-023-41801-w (PMC10545780; doi:10.1038/s41467-023-41801-w)
Supplement: Supplementary file 5 — Reporting Summary [file 41467_2023_41801_MOESM5_ESM.pdf]

Corresponding author(s): Lorenza Penengo
Last updated by author(s): Sep 7, 2023

Reporting Summary

Nature Portfolio wishes to improve the reproducibility of the work that we publish. This form provides structure for consistency and transparency in reporting. For further information on Nature Portfolio policies, see our Editorial Policies and the Editorial Policy Checklist.

Statistics

For all statistical analyses, confirm that the following items are present in the figure legend, table legend, main text, or Methods section.

n/a	Confirmed	
☐	☒	The exact sample size (*n*) for each experimental group/condition, given as a discrete number and unit of measurement
☐	☒	A statement on whether measurements were taken from distinct samples or whether the same sample was measured repeatedly
☐	☒	The statistical test(s) used AND whether they are one- or two-sided<br>*Only common tests should be described solely by name; describe more complex techniques in the Methods section.*
☒	☐	A description of all covariates tested
☐	☒	A description of any assumptions or corrections, such as tests of normality and adjustment for multiple comparisons
☐	☒	A full description of the statistical parameters including central tendency (e.g. means) or other basic estimates (e.g. regression coefficient) AND variation (e.g. standard deviation) or associated estimates of uncertainty (e.g. confidence intervals)
☐	☒	For null hypothesis testing, the test statistic (e.g. *F*, *t*, *r*) with confidence intervals, effect sizes, degrees of freedom and *P* value noted<br>*Give P values as exact values whenever suitable.*
☒	☐	For Bayesian analysis, information on the choice of priors and Markov chain Monte Carlo settings
☒	☐	For hierarchical and complex designs, identification of the appropriate level for tests and full reporting of outcomes
☒	☐	Estimates of effect sizes (e.g. Cohen's *d*, Pearson's *r*), indicating how they were calculated

Our web collection on statistics for biologists contains articles on many of the points above.

Software and code

Policy information about availability of computer code

Data collection	Immunofluorescence Images were acquired on a Leica DM6 B microscope. For quantitative image-based cytometry, images were converted to file system suitable for analysis with Olympus ScanR Image analysis software (version 3.0.1). The qPCR was run on a LightCycler® 480 II (Roche).<br>Mass spectrometry: Data acquisition was performed using data-dependent operation mode. Full-scan MS spectra (350–1’500 m/z) were acquired at a resolution of 120’000 at 200 m/z using Easy Spray Ion Source with spray voltage set to 2.3kV. MS/MS data were acquired using higher energy collision dissociation (HCD) fragmentation.<br>FACS analysis: For the cell death assay, Annexin V and PI staining were acquired on an Attune NxT Flow Cytometer (Thermo Fisher Scientific). For cell sorting, FACS (BD FACSAria™ III Cell Sorter) with a 488 nm argon ion laser based on their GFP fluorescence (using BD FACSDiva 9.0.1 software) was used.<br>Clonogenic assay: The colonies detected were fixed, stained with Brilliant Blue R and subsequently analyzed with the Gel-counter by Oxford Optronix and appertaining Software (version 1.1.2.0).
Data analysis	DNA fiber assay: Images were analyzed using ImageJ software (NIH). The quantification of RAD51 foci were exported and analyzed using Spotfire data visualization software (TIBCO, version 7.0.1).<br>Mass spectrometry: Raw data were searched by Mascot search engine (Matrix Science) against the human proteome database (UniProt entry 9606, taxonomy, 20190709), using cysteine carbamidomethylation as a fixed protein modification. Variable modifications consisted of methylation, oxidation, acetylation, deamidation and di-glycine addition on lysine residues. Precursor mass tolerance was set to 10 ppm and a maximum of two missed cleavages was allowed. Raw data were converted to Mascot Generic Format (MGF) using Proteome Discoverer, v1.4 (Thermo Fisher Scientific, Bremen, Germany) using the automated rule based converter control 71. Data processing was performed using Scaffold software (version 5.1, Proteome Software Inc., Portland, OR, USA). Protein identifications were accepted if they scored over 95% probability. Protein and peptide thresholds were set at 1% and 0.1% FDR, respectively, and a minimum number of two identified peptides for

each protein was allowed. The mass spectrometry proteomics data were handled using the local data management system B-Fabric. FACS analysis for the cell death assay, Annexin V and PI staining were analyzed using FlowJo software V.10.7.2 (FlowJo).

For manuscripts utilizing custom algorithms or software that are central to the research but not yet described in published literature, software must be made available to editors and reviewers. We strongly encourage code deposition in a community repository (e.g. GitHub). See the Nature Portfolio guidelines for submitting code & software for further information.

Data

Policy information about availability of data

All manuscripts must include a data availability statement. This statement should provide the following information, where applicable:

- Accession codes, unique identifiers, or web links for publicly available datasets
- A description of any restrictions on data availability
- For clinical datasets or third party data, please ensure that the statement adheres to our policy

The mass spectrometry proteomics data have been deposited to the ProteomeXchange Consortium via the PRIDE73 partner repository with the dataset identifier PXD045154. Data plotted in the mass spectrometry graph are presented in the Supplementary Data 1. Further information and requests for reagents and resources should be directed to the corresponding author. Source data are provided with this paper.

Research involving human participants, their data, or biological material

Policy information about studies with human participants or human data. See also policy information about sex, gender (identity/presentation), and sexual orientation and race, ethnicity and racism.

Reporting on sex and gender	N/A
Reporting on race, ethnicity, or other socially relevant groupings	N/A
Population characteristics	N/A
Recruitment	N/A
Ethics oversight	N/A

Note that full information on the approval of the study protocol must also be provided in the manuscript.

Field-specific reporting

Please select the one below that is the best fit for your research. If you are not sure, read the appropriate sections before making your selection.

☒ Life sciences ☐ Behavioural & social sciences ☐ Ecological, evolutionary & environmental sciences

For a reference copy of the document with all sections, see nature.com/documents/nr-reporting-summary-flat.pdf

Life sciences study design

All studies must disclose on these points even when the disclosure is negative.

Sample size	Sample size for all experiments shown (DNA fibers, n>100 in 2 or more independent experiments for IdU/CldU ratio or tract lengths, QIBC, >500 in 3 or more independent experiments) was chosen to obtain statistical power, in conformity to accepted standard sample size in a number of previous publications using these approaches. Mijic et al., Nat Commun., DOI: 10.1038/s41467-017-01164-5; Vujanovic et al., Mol Cell, DOI: 10.1016/j.molcel.2017.08.010; Andrs et al., Nat Commun., doi.org/10.1038/s41467-023-37341-y.
Data exclusions	No data were excluded from any of the analyses.
Replication	For all experiments, the number of biological replicates is indicated in the figure legends or in the Methods
Randomization	Experiments were not randomized. We were working with asynchronously cycling cell populations or individual DNA replication molecules from these cell populations. Hence further randomization or covariates management were not necessary for our approaches.
Blinding	Individual repetitions for DNA fiber analysis were blinded to the investigators.<br>Data collection and analysis related to the QIBC was conducted using unbiased image acquisition and analysis software, no other blinding was applied.

Reporting for specific materials, systems and methods

We require information from authors about some types of materials, experimental systems and methods used in many studies. Here, indicate whether each material, system or method listed is relevant to your study. If you are not sure if a list item applies to your research, read the appropriate section before selecting a response.

Materials & experimental systems

n/a	Involved in the study
☐	☒ Antibodies
☐	☒ Eukaryotic cell lines
☒	☐ Palaeontology and archaeology
☐	☒ Animals and other organisms
☒	☐ Clinical data
☒	☐ Dual use research of concern
☒	☐ Plants

Methods

n/a	Involved in the study
☒	☐ ChIP-seq
☐	☒ Flow cytometry
☒	☐ MRI-based neuroimaging

Antibodies

Antibodies used

BRCA1 Santa Cruz Biotechnology sc-6954 AB_626761
BRCA2 Millipore OP95-100ug AB_213443
FLAG Sigma F7425 AB_439687
GAPDH Millipore MAB374 AB_2107445
ISG15 This paper N/A N/A
ISG15 K.P. Knobeloch Institute of Neuropathology, Freiburg, Germany N/A N/A
ISG15 Santa Cruz Biotechnology sc-166755 AB_2126308
Lamin A Sigma-Aldrich L1293 AB_532254
MYC Santa Cruz Biotechnology sc-40 AB_627268
PCNA Santa Cruz Biotechnology sc-56 AB_628110
TOP1 Novus Biologicals NBP1-90365 AB_11023377
TRIM25 This paper N/A N/A
TRIM25 Abcam ab167154 AB_2721902
Tubulin Sigma-Aldrich T5168 AB_477579
Vinculin Thermo Fisher Scientific 700062 AB_2532280

Validation

Specificities of the antibodies against BRCA1, BRCA2, ISG15, TOP1 and TRIM25 were confirmed by protein knockdown with specific siRNA for the corresponding target and by using cell lines carrying either deletion or mutations in BRCA1, BRCA2, ISG15 and TRIM25. Specificities of the antibodies against GAPDH, FLAG, Vinculin, PCNA, Tubulin, MYC and Lamin A were validated by the manufacturer for western blots as indicated on manufacturer website (see below).

GAPDH: https://www.merckmillipore.com/CH/de/product/Anti-Glyceraldehyde-3-Phosphate-Dehydrogenase-Antibody-clone-6C5,MM_NF-MAB374?ReferrerURL=https%3A%2F%2Fwww.google.com%2F
FLAG: https://www.sigmaaldrich.com/CH/en/product/sigma/f7425
Lamin A: https://www.sigmaaldrich.com/CH/de/product/sigma/sab4501765?gclid=EAIaIQobChMI442Cju6DgAMVToZoCR1HWQnEEAAYASAAEgIFpfD_BwE&gclsrc=aw.ds
MYC: https://www.scbt.com/p/c-myc-antibody-9e10
PCNA: (PC10) mouse monoclonal (sc56, Santa Cruz Biotechnology); https://www.scbt.com/p/pcna-antibody-pc10?requestFrom=search
Tubulin: https://www.sigmaaldrich.com/CH/en/product/sigma/t5168
Vinculin. https://www.thermofisher.com/antibody/product/Vinculin-Antibody-clone-42H89L44-Recombinant-Monoclonal/700062

Eukaryotic cell lines

Policy information about cell lines and Sex and Gender in Research

Cell line source(s)

U2OS (HTB-96), HEK293T (CRL-11268), Capan-1 (HTB-79) , MCF-7 (HTB-22) were from ATCC. U2OS T-REx FLAG-ISG15 was prepared in Penengo lab (doi.org/10.1083/jcb.202002175). MDA-MB 436 BRCA1-/- and BRCA1+/+ (reconstituted) cells were kindly gifted by Neil Johnson; Brca2+/+ and Brca2-/- mouse mammary tumor cells (KB2P 1.21) have been previously described (Jonkers, J. et al. Nat Genetics 2001). Mouse embryonic stem cells (mESCs) and PL2F7 were provided by Shyam Sharan and generated as in Kuznetsov et al., Nat Med 2008. Trim25+/+ and Trim25-/- MEFs were kindly gifted by Satoshi Inoue (Tokyo Metropolitan Institute of Gerontology, University of Tokyo, Japan). Ube1L+/+ and Ube1L-/- MEFs were kindly gifted by Dong-Er Zhang (Moores Cancer Center, University of California, San Diego, USA). Isg15+/+ and Isg15-/- MEFs were kindly gifted by Klaus-Peter Knobeloch (Institute of Neuropathology, University Clinic Freiburg, Germany).

Authentication

None of these cell lines were authenticated in house for this manuscript.

Mycoplasma contamination

We routinely do mycoplasma testing on our cell lines and the cell lines used in this study tested negative.

Commonly misidentified lines
(See ICLAC register)

No commonly misidentified lines were used in this study.

Animals and other research organisms

Policy information about studies involving animals; ARRIVE guidelines recommended for reporting animal research, and Sex and Gender in Research

Laboratory animals	*For laboratory animals, report species, strain and age OR state that the study did not involve laboratory animals.*
Wild animals	*Provide details on animals observed in or captured in the field; report species and age where possible. Describe how animals were caught and transported and what happened to captive animals after the study (if killed, explain why and describe method; if released, say where and when) OR state that the study did not involve wild animals.*
Reporting on sex	*Indicate if findings apply to only one sex; describe whether sex was considered in study design, methods used for assigning sex. Provide data disaggregated for sex where this information has been collected in the source data as appropriate; provide overall numbers in this Reporting Summary. Please state if this information has not been collected. Report sex-based analyses where performed, justify reasons for lack of sex-based analysis.*
Field-collected samples	*For laboratory work with field-collected samples, describe all relevant parameters such as housing, maintenance, temperature, photoperiod and end-of-experiment protocol OR state that the study did not involve samples collected from the field.*
Ethics oversight	*Identify the organization(s) that approved or provided guidance on the study protocol, OR state that no ethical approval or guidance was required and explain why not.*

Note that full information on the approval of the study protocol must also be provided in the manuscript.

Flow Cytometry

Plots

Confirm that:

☒ The axis labels state the marker and fluorochrome used (e.g. CD4-FITC).

☒ The axis scales are clearly visible. Include numbers along axes only for bottom left plot of group (a 'group' is an analysis of identical markers).

☒ All plots are contour plots with outliers or pseudocolor plots.

☒ A numerical value for number of cells or percentage (with statistics) is provided.

Methodology

Sample preparation	BRCA1-deficient MDA-MB-436 cells were plated (3x105) in 6 cm plates 24 h prior to siRNA knockdown. siLuc or siISG15 (40 nM) were transfected. Seventy-two or 120 h after siRNA transfection, cells were collected by trypsinization. Using 700'000 cells per conditions, Annexin V and PI staining was performed using eBioscienceTM Annexin V Apoptosis Detection Kit FITC (Invitrogen, 88-8005-74) according to manufacturer's protocol. In short, cells were washed once in PBS, then once in Binding Buffer. Cells were resuspended in µL Binding Buffer and Annexin V FITC was added; cells were incubated for 15 min at room temperature and subsequently washed in Binding Buffer. Cells were resuspended in Binding Buffer and PI staining solution was added.
Instrument	Attune NxT Flow Cytometer (Thermo Fisher Scientific)
Software	FlowJo software V.10.7.2 (FlowJo)
Cell population abundance	the entire population of cells was analysed for morphological parameters (FSC, SSC) and for staining with PI and Annexin V
Gating strategy	No gating strategy was applied. All cells were analysed for all parameters in all samples

☐ Tick this box to confirm that a figure exemplifying the gating strategy is provided in the Supplementary Information.
